# Factors influencing health-promoting lifestyles in young and middle-aged adults with metabolic risk factors: a network analysis

**DOI:** 10.1186/s12889-025-25192-9

**Published:** 2025-11-07

**Authors:** Qun Wang, Sek Ying Chair, Weixiang Luo, Siyi Zhong, Zehao Huang

**Affiliations:** 1 School of Nursing, Shenzhen University, Shenzhen, China; 2 The Nethersole School of Nursing, Faculty of Medicine, The Chinese University of Hong Kong, Hong Kong, China; 3 Department of Nursing, Shenzhen People’s Hospital, Shenzhen, China; 4 School of Public Health, Shenzhen University, Shenzhen, China

**Keywords:** Metabolic risk factors, Health-promoting lifestyles, Predictors, Network analysis

## Abstract

**Background:**

Metabolic dysfunction is prevalent among young and middle-aged adults and impairs their health. Adopting healthy lifestyles is essential for preventing and managing these issues. By determining modifiable factors that influence health-promoting lifestyles, we can gain valuable insights for developing targeted lifestyle interventions. Therefore, this study aims to identify factors contributing to health-promoting lifestyles among young and middle-aged adults with metabolic risk factors.

**Methods:**

A cross-sectional study design was adopted. Data were obtained from 402 young and middle-aged adults with metabolic risk factors, recruited via convenience sampling from community health centers in China between October 2022 and April 2024. All participants were measured for knowledge (MetS Knowledge Scale), self-efficacy (Self-Rated Abilities for Health Practices Scale), social support (Social Support Rating Scale), depression (Hospital Anxiety and Depression Scale-Depression), anxiety (Hospital Anxiety and Depression Scale-Anxiety), and health-promoting lifestyles (Health-Promoting Lifestyle Profile II). The impact of self-efficacy, knowledge, social support, depression, anxiety on health-promoting lifestyles was estimated using regression and network analysis via SPSS and R program, respectively.

**Results:**

The mean scores for knowledge, social support, depression, anxiety, self-efficacy, and health-promoting lifestyles are 28.01 (SD = 12.31), 39.81 (SD = 7.52), 4.60 (SD = 3.25), 5.22 (SD = 3.50), 65.92 (SD = 22.43), and 58.25 (SD = 7.52), respectively. After controlling the covariates, the regression analysis showed that knowledge (β = 0.176, *P* < 0.001) and self-efficacy (β = 0.299, *P* < 0.001) are crucial predictors of dietary behaviors. Social support (β = 0.099, *P* = 0.027), depression (β=-0.181, *P* < 0.001), and self-efficacy (β = 0.434, *P* < 0.001) had significant effects on stress management. In addition, self-efficacy (β = 0.485, *P* < 0.001) plays an important role in physical activity. The network analysis further indicated that self-efficacy for psychological well-being serves as a core and bridge factor influencing health-promoting lifestyles among young and middle-aged adults with metabolic risk factors.

**Conclusions:**

Self-efficacy, knowledge, social support, and depression are crucial factors for predicting health-promoting lifestyles in young and middle-aged adults with metabolic risk factors. Moreover, self-efficacy for psychological well-being is the key component that need to be prioritized when developing intervention programs to promote meaningful lifestyle changes.

**Supplementary Information:**

The online version contains supplementary material available at 10.1186/s12889-025-25192-9.

## Introduction

Metabolic dysfunction contributes to the onset of various chronic diseases, including chronic kidney disease, diabetes, and heart disease, and leads to increased mortality rates [1–5]. It was reported that common metabolic risk factors such as abdominal obesity, high blood glucose, dyslipidemia, and hypertension account for more than a quarter of global disability-adjusted life years [5, 6]. Importantly, recent studies have revealed a conspicuous global burden of these risk factors among younger populations [7, 8]. It has been reported that the prevalence of metabolically unhealthy obesity among young and middle-aged adults in China is 3.07% and 5.6%, respectively [9]. Weight gain during young and middle adulthood significantly increases the risk of major chronic diseases and reduces the likelihood of healthy aging [10]. Recent data indicates that the prevalence of hypertension in China has increased to 12.8% among young adults and 37.4% in middle-aged group [11]. The prevalence of total diabetes is 3.1% for young Chinese adults and 8.7% for middle-aged individuals [12]. A systematic review reported a pool prevalence of dyslipidemia of 40.1% in young adults and 45.2% among middle-aged adults in China [13]. Lifestyle interventions are crucial for preventing and managing these risk factors [14, 15], focusing primarily on balanced nutrition, regular physical activity, and stress management [14]. Therefore, to develop effective lifestyle interventions for young and middle-aged adults with metabolic risk factors, it is essential to determine the modifiable predictors of health-promoting lifestyles.

Our previous research revealed that patients with metabolic risk factors often lacked sufficient knowledge about the diagnosis and complications of metabolic syndrome, as well as information on how to prevent it [16]. Addressing these issues may facilitate better adoption of health-promoting lifestyles among young and middle-aged adults with metabolic risk factors [17, 18]. Self-efficacy in executing health behaviors is one of the essential predictors of health-promoting lifestyles [19]. A prior investigation demonstrated that enhancing self-efficacy contributes to improved daily physical activity and eating behaviors among adults with or at high risk of metabolic syndrome, while evidence of its impact on stress management is scarce [19]. Moreover, social support serves as an important element in promoting healthy lifestyles [20], with research indicating that providing support has a direct effect on healthy eating in patients with metabolic syndrome [18]. However, evidence for its impact on other health-promoting behaviors in this population is limited. Adults with metabolic risk factors such as hyperglycemia and increased triglycerides, as well as decreased high-density lipoprotein, have a higher risk of developing depression and anxiety [21]. Depression is significantly negatively associated with stress management in adults with metabolic syndrome, while evidence regarding its relationship with physical activity and dietary behaviors remains inconclusive [22]. Anxiety may correlate with health-promoting lifestyles in this clinical cohort; however, current evidence is sparse [23]. Due to the insufficient evidence on the predictors of health-promoting lifestyles among young and middle-aged adults with metabolic risk factors, further investigation is necessary.

Traditional methods, such as regression analysis, are limited in their ability to elucidate the interrelationships between multiple variables. Network analysis allows for the estimation of complex patterns of relationships, and examining the network structure can uncover its core characteristics, offering valuable insights for developing targeted lifestyle interventions [24]. Therefore, this study aimed to explore the predictors of health-promoting lifestyles among young and middle-aged adults with metabolic risk factors using both regression and network analysis.

## Methods

### Study design and participants

This was a cross-sectional study conducted in Shenzhen, China, from October 2022 to April 2024. The Strengthening of Reporting of Observational Studies in Epidemiology checklist for cross-sectional studies was followed [25]. We consecutively enrolled young and middle-aged adults with metabolic risk factors using convenience sampling. The inclusion criteria were as follows: (1) the presence of at least one metabolic syndrome risk factor (central obesity, elevated triglycerides, reduced high-density lipoprotein cholesterol, elevated blood pressure, and hyperglycemia); (2) age between 18 and 59; (3) ability to communicate in Chinese; (4) ability to complete the questionnaires; (5) ability to provide written consent. Patients were excluded if they met any of the following criteria: (1) a diagnosis of diabetes, coronary heart disease, or stroke; (2) clinical diagnoses of psychiatric disorders; or (3) pregnancy or breastfeeding. To achieve sufficient statistical power, a minimum sample size of 250 to 350 participants is needed if the network consists of 20 nodes or fewer [26]. In this study, data from 402 valid questionnaires were used.

### Data collection and outcome measures

Data collection was conducted in six community health centers (CHC) in the Baoan, Nanshan, and Longhua districts of Shenzhen. Online and offline posters were distributed by the healthcare professionals of six CHCs. Those who responded to the invitation were screened by the research team. An introduction letter with the study objectives and procedures was given to potential participants. After receiving the written informed consent, the research team scheduled time for data collection. Trained investigators administered data collection in the CHC. The participants were recommended to complete the questionnaires by themselves. The trained investigators also assisted if necessary. The Chinese versions of the instruments have been validated.

#### Knowledge

The knowledge of MetS was evaluated using the 10-item MetS Knowledge Scale, which includes three domains, namely definition and diagnosis of MetS (five items), complications of MetS (two items), and prevention of MetS (three items). Each item was scored as 10 for a correct response and 0 for an incorrect response or “do not know”. The total score, calculated as the sum of all item scores, ranges from 0 to 100, with higher scores implying a greater understanding of metabolic syndrome [16, 27]. The Cronbach’s α for this study was 0.716.

#### Self-efficacy

The 28-item Self-Rated Abilities for Health Practices Scale was adopted to assess self-efficacy. It contains four subscales (seven items each): exercise, nutrition, responsible health practice, and psychological well-being. Each item was rated on a 5-point Likert scale, ranging from 0 (not at all) to 4 (completely). Total score is the sum of all item scores, ranging from 0 to 112, with higher scores reflecting greater self-efficacy in health practices [28, 29]. The Cronbach’s α for this study was 0.950.

#### Social support

The 10-item Social Support Rating Scale was used to measure social support. The scale consists of three domains: objective support (three items), subjective support (four items), and support utilization (three items). The items on the scale include single-choice (a 4-point Likert scale) and multiple-choice options. Total score is the sum of all item scores, with higher scores indicating higher levels of social support [30]. The Cronbach’s α for this study was 0.617.

#### Depression

The 7-item Hospital Anxiety and Depression Scale-Depression was employed to assess depression. A 4-point Likert scale was used for scoring each item, ranging from 0 to 3. Total scores are the sum of all item scores, ranging from 0 to 21, with higher scores suggesting higher levels of depression [31]. The Cronbach’s α for this study was 0.662.

#### Anxiety

The 7-item Hospital Anxiety and Depression Scale-Anxiety was used to evaluate anxiety. A 4-point Likert scale was used for scoring each item, ranging from 0 to 3. Total scores are the sum of all item scores, ranging from 0 to 21, with higher scores suggesting higher levels of anxiety [31]. The Cronbach’s α for this study was 0.774.

#### Health-promoting lifestyles

Three subscales of Health-Promoting Lifestyle Profile II, including nutrition (9 items), physical activity (8 items), and stress management (8 items), were adopted to measure the practice of health-promoting lifestyles. Participants rated on a four-point Likert scale, ranging from 1 (never) to 4 (routinely). The total scores for each subscale were calculated as the means of the responses to all items within that subscale. A higher score indicates better performance in health-promoting lifestyles [17, 32]. The Cronbach’s α for this study was 0.836.

#### Sociodemographic information

The sociodemographic information includes age, gender, marital status, employment status, educational level, monthly income, and smoking and drinking history.

### Data analysis

The statistical analyses were conducted using IBM SPSS version 27.0. A two-sided *p* < 0.05 is considered statistically significant. The statistical assumption of the normality of continuous variables was examined by quantile-quantile plots and skewness and kurtosis statistics [33]. Normally distributed continuous variables were presented in means and standard deviation (SDs). All the categorical variables were presented as frequency and percentage. Independent t-tests, one-way analysis of variance, or Pearson’s correlation analysis were used to identify potential predictors of health-promoting lifestyles. Hierarchical linear regression analyses were executed to test the relationships between study variables and health-promoting lifestyles while controlling significant sociodemographic variables that correlated with health-promoting lifestyles. We assessed multicollinearity using tolerance and variance inflation factors (VIF), with acceptable criteria set at a tolerance greater than 0.1 and a VIF less than 10 [34].

Network analysis was performed using RStudio 2025.05.0 + 496 to analyze the relationships between the study variables. The network was estimated using the Gaussian Graphical Model with the EBICglasso algorithm and visualized with the “qgraph” package. In the network, nodes represent study variables or their domains, while edges denote the links between the nodes. The thickness of each edge indicates the strength of the relationship, with blue representing positive relationships and red indicating negative correlations. To identify core nodes in the network, centrality analysis was conducted using two centrality indicators: strength and closeness. Strength reflects the extent of direct connections a node has with others, while closeness assesses indirect connections. A node with the highest values of strength and closeness was deemed the core node. Moreover, bridge strength, defined as the total sum of the absolute values of the connections between a given node and all nodes outside its own cluster, was calculated to identify the bridge node that plays a crucial role in connecting different clusters within the network using the “networktools” package. We adopted “bootnet” package to estimate the accuracy and stability of the network. The accuracy of edge weights was evaluated by computing the 95% confidence intervals using a nonparametric bootstrap with 1,000 samples. The stability of the network was estimated by calculating the correlation stability coefficient using a case-dropping subset bootstrap with 1000 bootstrap samples. Cut-off values of 0.25 were considered indicative of acceptable stability, while values of 0.50 were deemed as good stability [24, 35]. Bootstrapped difference tests were conducted to determine if there were differences in node strength and edge weight using the “NetworkComparisonTest” package.

## Results

### Participant characteristics

Among the recruited subjects, ages ranged from 20 to 59 years, with a mean age of 46.93 years. The majority of participants were male (70.6%), married (89.3%), and employed. The mean scores for knowledge, social support, depression, anxiety, self-efficacy, and health-promoting lifestyles are 28.01 (SD = 12.31), 39.81 (SD = 7.52), 4.60 (SD = 3.25), 5.22 (SD = 3.50), 65.92 (SD = 22.43), and 58.25 (SD = 7.52), respectively. Table 1 shows the details of participant characteristics.

**Table 1:** Participant characteristics (*n* = 402)

Variables	Mean ± SD/*N* (%)
Age (year) (Range: 20 ~ 59)	46.93 ± 9.03
Gender	
Male	284 (70.6)
Female	118 (29.4)
Marital status	
Married	359 (89.3)
Others	43 (10.7)
Employment status	
Employed	309 (76.9)
Unemployed	93 (23.1)
Educational level	
Primary school or less	157 (39.1)
Secondary school	119 (29.6)
Tertiary school or above	126 (31.3)
Monthly income (CNY)	
<2500	68 (16.9)
2500–5000	122 (30.3)
5000–10,000	114 (28.4)
≥ 10,000	98 (24.4)
Smoking history	
Smoking	89 (22.1)
Ex-smoker	39 (9.7)
No	274 (68.2)
Drinking history	
Regular drinking	44 (10.9)
Ex-drinker	21 (5.2)
Occasional drinking	184 (45.8)
No	153 (38.1)
Knowledge	28.01 ± 21.31
Social support	39.81 ± 7.52
Depression	4.60 ± 3.25
Anxiety	5.22 ± 3.50
Self-efficacy	65.92 ± 22.43
Health-promoting lifestyles	58.25 ± 7.52

### Potential predictors of health-promoting lifestyles

As presented in Table 2, we found significant differences in the nutritional domain of health-promoting lifestyles concerning gender, marital status, drinking history, age, knowledge, social support, depression, anxiety, and self-efficacy. The results also showed significant differences in stress management with respect to educational level, knowledge, social support, depression, anxiety, and self-efficacy. Additionally, significant differences in physical activity were noted regarding educational level, knowledge, depression, and self-efficacy.

**Table 2:** Potential predictors of health-promoting lifestyles

Variables		HPLP II _N	HPLP II _S	HPLP II _PA
		Mean ± SD	Mean ± SD	Mean ± SD
Gender	Male	22.20 ± 3.82	18.99 ± 4.39	16.73 ± 4.50
	Female	23.25 ± 3.97	19.53 ± 4.32	16.25 ± 4.69
	*t*	−2.481	−1.138	0.974
	*P*	0.014	0.256	0.330
Marital status	Married	22.65 ± 3.89	19.19 ± 4.43	16.63 ± 4.53
	Others	21.40 ± 3.76	18.84 ± 3.88	16.28 ± 4.87
	*t*	−2.001	−0.499	−0.472
	*P*	0.046	0.618	0.637
Employment status	Employed	22.34 ± 3.85	18.96 ± 4.35	16.42 ± 4.54
	Unemployed	23.09 ± 3.97	19.77 ± 4.41	17.16 ± 4.59
	*t*	−1.626	−1.569	0.980
	*P*	0.105	0.117	0.168
Educational level	Primary school or less	22.29 ± 3.67	18.42 ± 4.43	15.55 ± 4.30
	Secondary school	22.37 ± 4.14	19.20 ± 4.07	17.21 ± 4.30
	Tertiary school or above	22.93 ± 3.91	20.02 ± 4.44	17.29 ± 4.90
	*F*	1.067	4.755	6.846
	*P*	0.345	0.009	0.001
Monthly income (CNY)	< 2500	22.75 ± 4.80	18.96 ± 4.34	15.79 ± 4.85
	2500–5000	22.45 ± 3.86	18.66 ± 4.64	16.11 ± 4.44
	5000–10,000	22.72 ± 3.69	19.41 ± 4.03	17.27 ± 4.25
	≥ 10,000	22.18 ± 3.74	19.15 ± 4.37	16.94 ± 4.77
	*F*	0.434	1.018	2.193
	*P*	0.729	0.384	0.088
Smoking history	Smoking	21.69 ± 3.74	18.45 ± 4.30	15.75 ± 4.48
	Ex-smoker	23.05 ± 4.17	18.97 ± 4.56	16.79 ± 3.84
	No	22.70 ± 3.87	19.41 ± 4.36	16.83 ± 4.66
	*F*	2.744	1.646	1.935
	*P*	0.065	0.194	0.146
Drinking history	Regular drinking	20.48 ± 3.72	17.82 ± 4.12	15.86 ± 3.61
	Ex-drinker	23.57 ± 3.64	19.33 ± 3.89	17.71 ± 4.33
	Occasional drinking	22.77 ± 3.50	19.47 ± 4.33	16.95 ± 4.48
	No	22.65 ± 4.25	19.12 ± 4.52	16.21 ± 4.89
	*F*	5.004	1.719	1.544
	*P*	0.002	0.162	0.202
Age	*r*	0.171	0.042	0.054
	*P*	< 0.001	0.400	0.284
Knowledge	*r*	0.195	0.167	0.126
	*P*	< 0.001	< 0.001	0.012
Social support	*r*	0.181	0.220	0.084
	*P*	< 0.001	< 0.001	0.093
Depression	*r*	−0.246	−0.316	−0.202
	*P*	< 0.001	< 0.001	< 0.001
Anxiety	*r*	−0.117	−0.144	−0.032
	*P*	0.019	0.004	0.528
Self-efficacy	*r*	0.345	0.506	0.502
	*P*	< 0.001	< 0.011	< 0.001

*t* Independent t-tests, *F* one-way analysis of variance, *HPLP II* Health-Promoting Lifestyle Profile II, *N* nutrition, *S* Stress management, *PA* Physical activity

### Hierarchical linear regression analysis

No multicollinearity was detected in the present study, as tolerance values were above 0.1 and VIF values were below 10. After controlling the significant characteristics of participants, the results showed that knowledge (*β* = 0.176, *P* < 0.001) and self-efficacy (*β* = 0.299, *P* < 0.001) had significant effects on the nutritional domain of health-promoting lifestyles. We also observed significant predictive roles of social support (*β* = 0.099, *P* = 0.027), depression (*β*=−0.181, *P* < 0.001), and self-efficacy (*β* = 0.434, *P* < 0.001) on stress management. In addition, self-efficacy (*β* = 0.485, *P* < 0.001) significantly predicted physical activity (Table 3).

**Table 3:** Hierarchical linear regressions of factors affecting health-promoting lifestyles

	Variable	Step 1		Collinearity statistics		Step 2		Collinearity statistics	
		β	*P*	Tolerance	VIF	β	*P*	Tolerance	VIF
HPLP II_N	Gender	0.114	0.041	0.748	1.337	0.095	0.065	0.732	1.367
	Marital status	0.021	0.704	0.771	1.298	0.012	0.816	0.687	1.456
	Ex-drinker	0.167	0.004	0.711	1.406	0.169	0.001	0.705	1.419
	Occasional drinking	0.288	< 0.001	0.353	2.836	0.250	< 0.001	0.348	2.872
	No drinking	0.188	0.030	0.312	3.208	0.205	0.009	0.311	3.215
	Age	0.150	0.007	0.767	1.304	0.227	< 0.001	0.659	1.518
	Knowledge					0.176	< 0.001	0.772	1.295
	Social support					0.039	0.435	0.779	1.283
	Depression					−0.081	0.132	0.669	1.494
	Anxiety					−0.011	0.836	0.699	1.431
	Self-efficacy					0.299	< 0.001	0.795	1.257
	*R2 (ΔR2)*	0.075 (0.075)				0.249 (0.174)			
	*F*	5.334***				11.732***			
HPLP II_S	Secondary school	0.082	0.139	0.808	1.238	−0.029	0.550	0.734	1.363
	Tertiary school or above	0.170	0.002	0.808	1.238	0.020	0.358	0.576	1.736
	Knowledge					0.016	0.745	0.750	1.334
	Social support					0.099	0.027	0.890	1.124
	Depression					−0.181	< 0.001	0.697	1.434
	Anxiety					−0.008	0.875	0.699	1.431
	Self-efficacy					0.434	< 0.001	0.752	1.329
		0.023 (0.023)				0.305 (0.282)			
		4.755**				24.676***			
HPLP II_PA	Secondary school	0.166	0.003	0.808	1.238	0.056	0.262	0.742	1.347
	Tertiary school or above	0.177	0.001	0.808	1.238	0.018	0.749	0.603	1.660
	Knowledge					−0.040	0.414	0.764	1.308
	Depression					−0.081	0.074	0.917	1.091
	Self-efficacy					0.485	< 0.001	0.772	1.295
		0.033 (0.033)				0.262 (0.229)			
		6.846**				28.048***			

***p < 0.001, **p < 0.01, *VIF* Variance Inflation Factor, *HPLP II* Health-Promoting Lifestyle Profile II, *N* Nutrition, *S * Stress management, *PA * Physical activity

### Network analysis

**Fig. 1:**
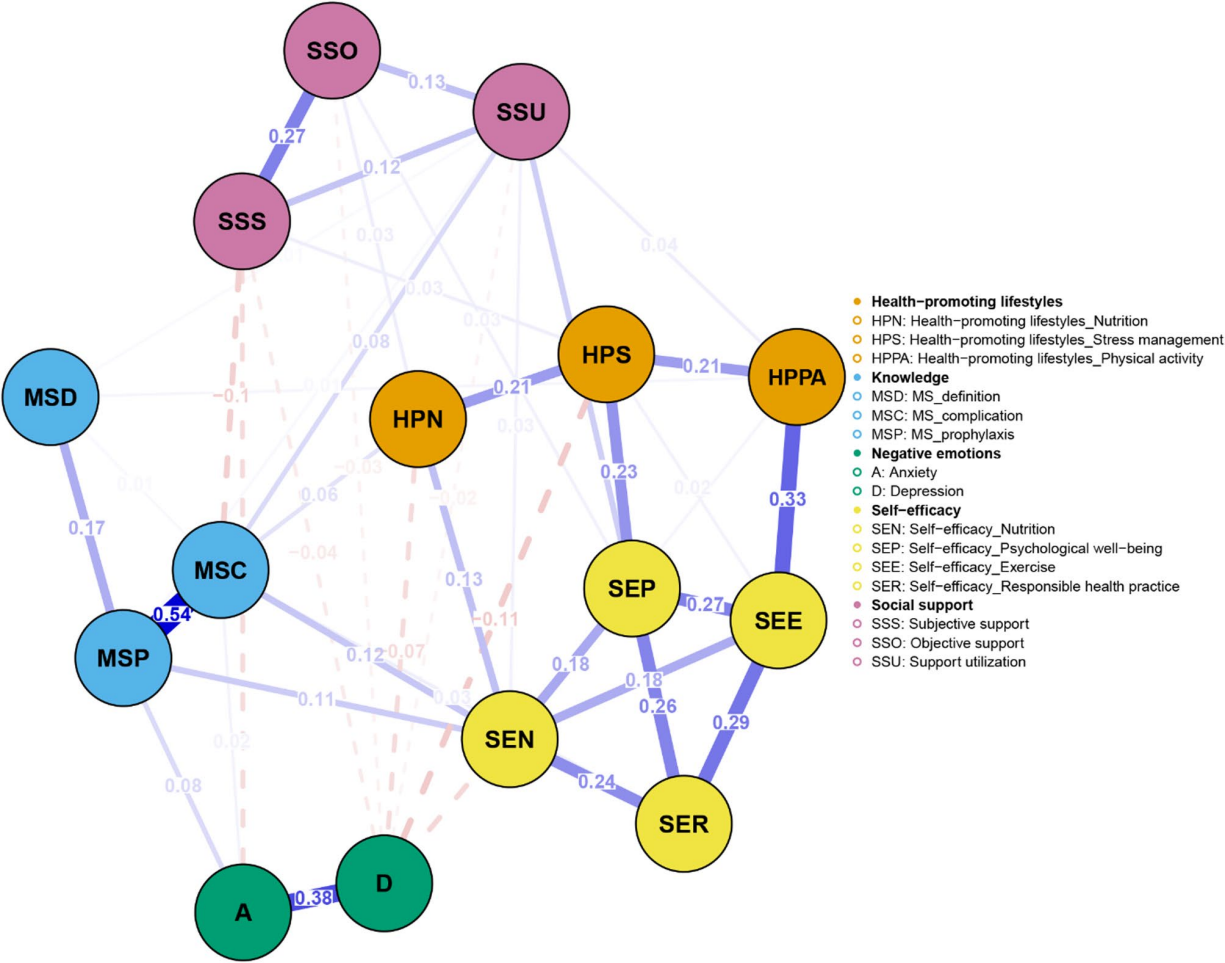
Network structure of health-promoting lifestyles and its influencing factors

**Fig. 2:**
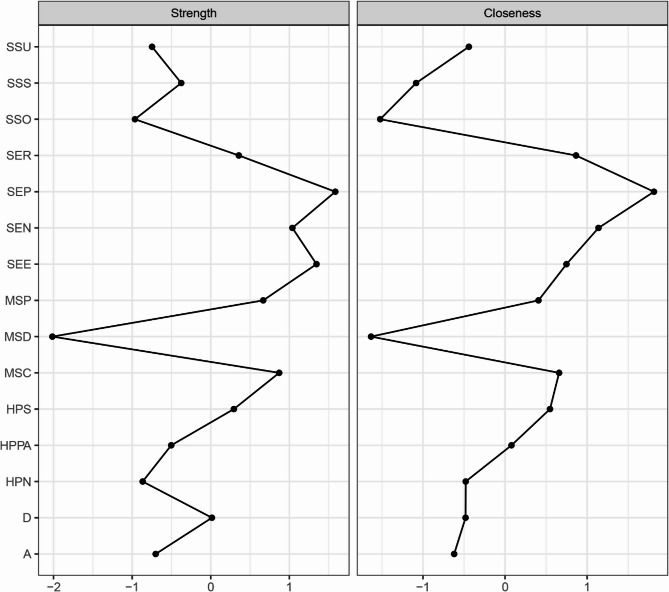
Standardized centrality indices of network (z-scores) Note: *SSU* Support utilization, *SSS* Subjective support, *SSO* Objective support, *SER* Self-efficacy_Responsible health practice, *SEP * Self-efficacy_Psychological well-being, *SEN* Self-efficacy_Nutrition, *SEE* Self-efficacy_Exercise, *MSP* MS_prophylaxis, *MSD* MS_definition, *MSC* MS_complication, *HPS* Health-promoting lifestyles_Stress management, *HPPA* Health-promoting lifestyles_Physical activity, *HPN* Health-promoting lifestyles_Nutrition, *D* Depression, *A* Anxiety

**Fig. 3:**
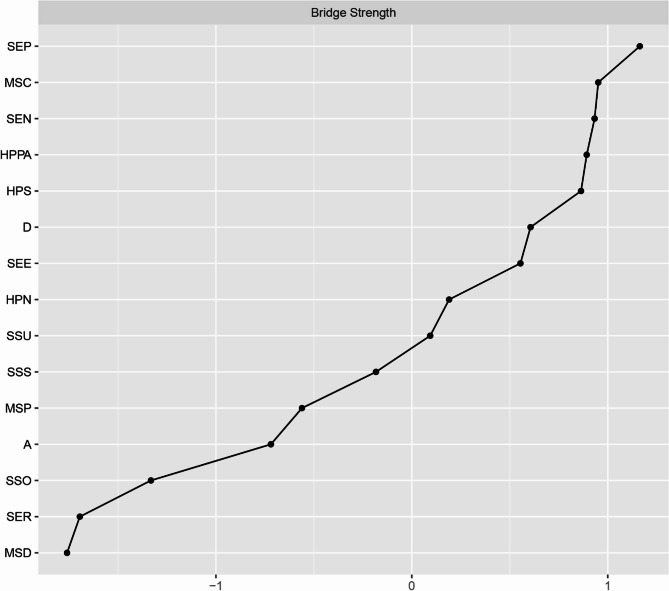
Standardized bridge centrality indices of network (z-scores). Note: *SEP* Self-efficacy_Psychological well-being, *MSC* MS_complication, *SEN* Self-efficacy_Nutrition *HPPA* Health-promoting lifestyles_Physical activity, *HPS* Health-promoting lifestyles_Stress management, *D* Depression, *SEE* Self-efficacy_Exercise, *HPN* Health-promoting lifestyles_Nutrition, *SSU* Support utilization, *SSS* Subjective support, *MSP* MS_prophylaxis, *A* Anxiety, *SSO* Objective support, *SER* Self-efficacy_Responsible health practice, *MSD * MS_definition

**Fig. 4:**
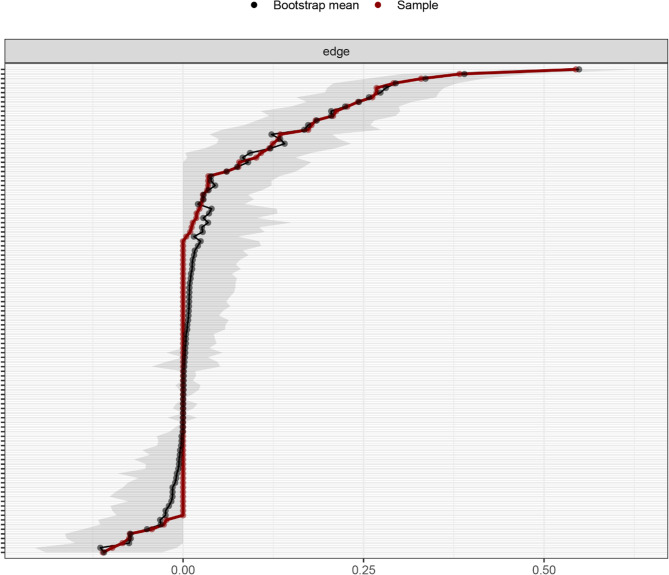
Bootstrap 95% confidence intervals of the edge weight

**Fig. 5:**
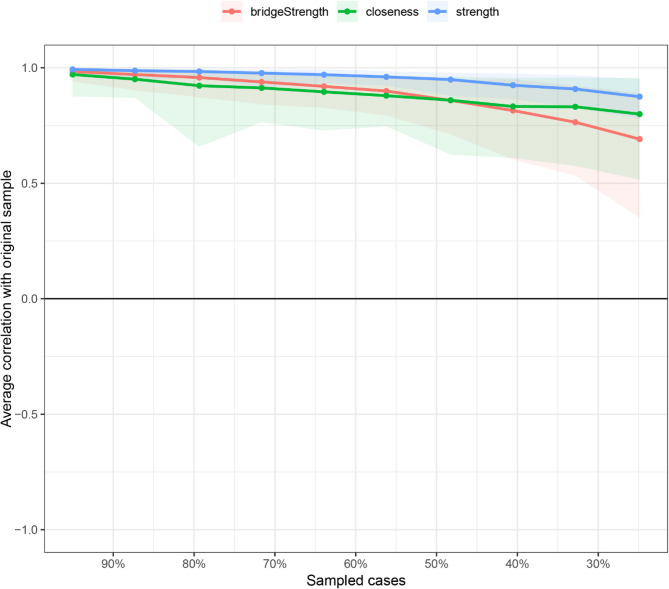
Correlation stability coefficient for strength, closeness, and bridge strength

**Fig. 6:**
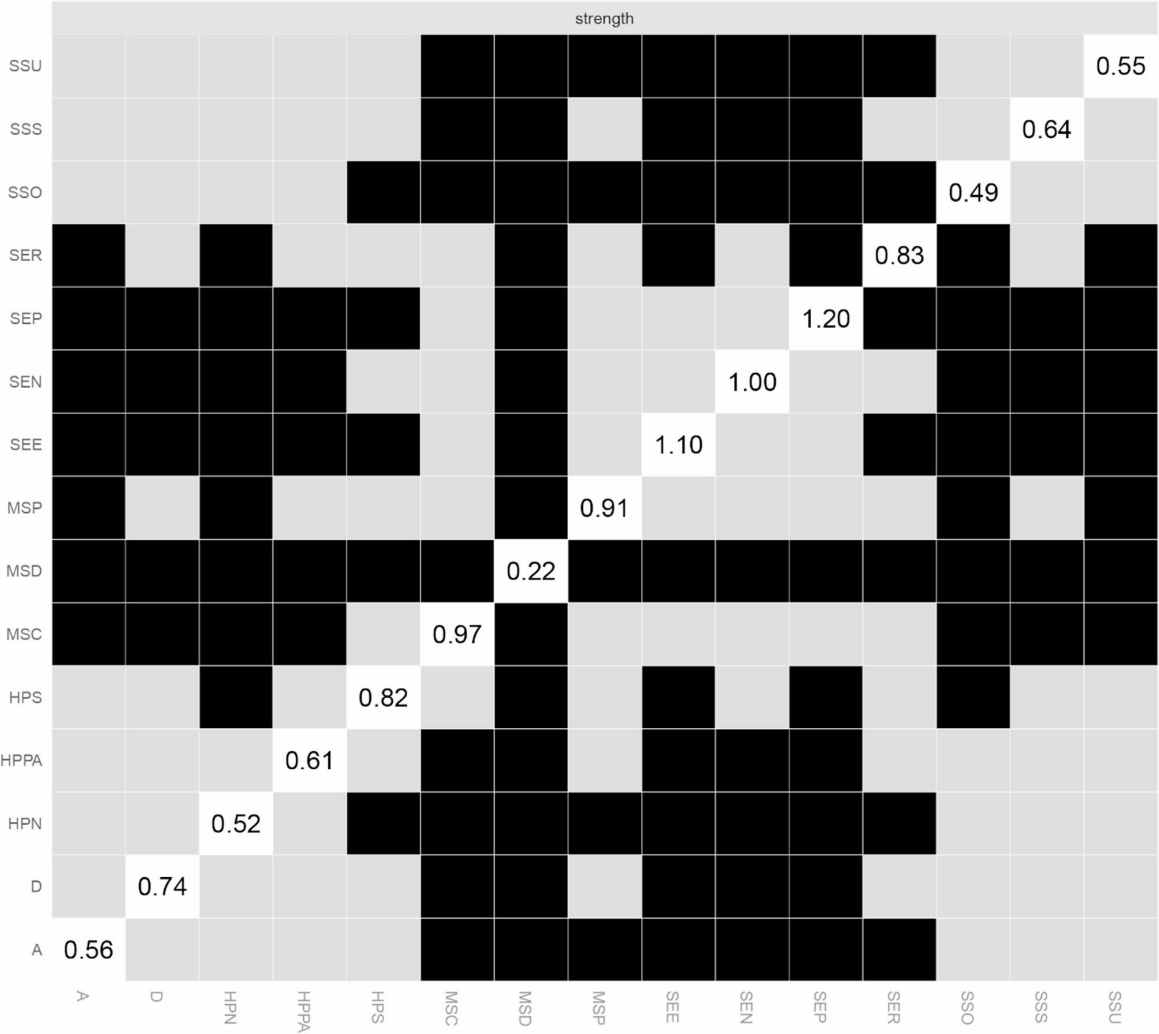
Estimation of node strength difference by bootstrapped difference test Note: Gray boxes indicate no significant differences between nodes, while black boxes represent nodes that exhibit significant differences (α = 0.05); White boxes display the values of node strength

**Fig. 7:**
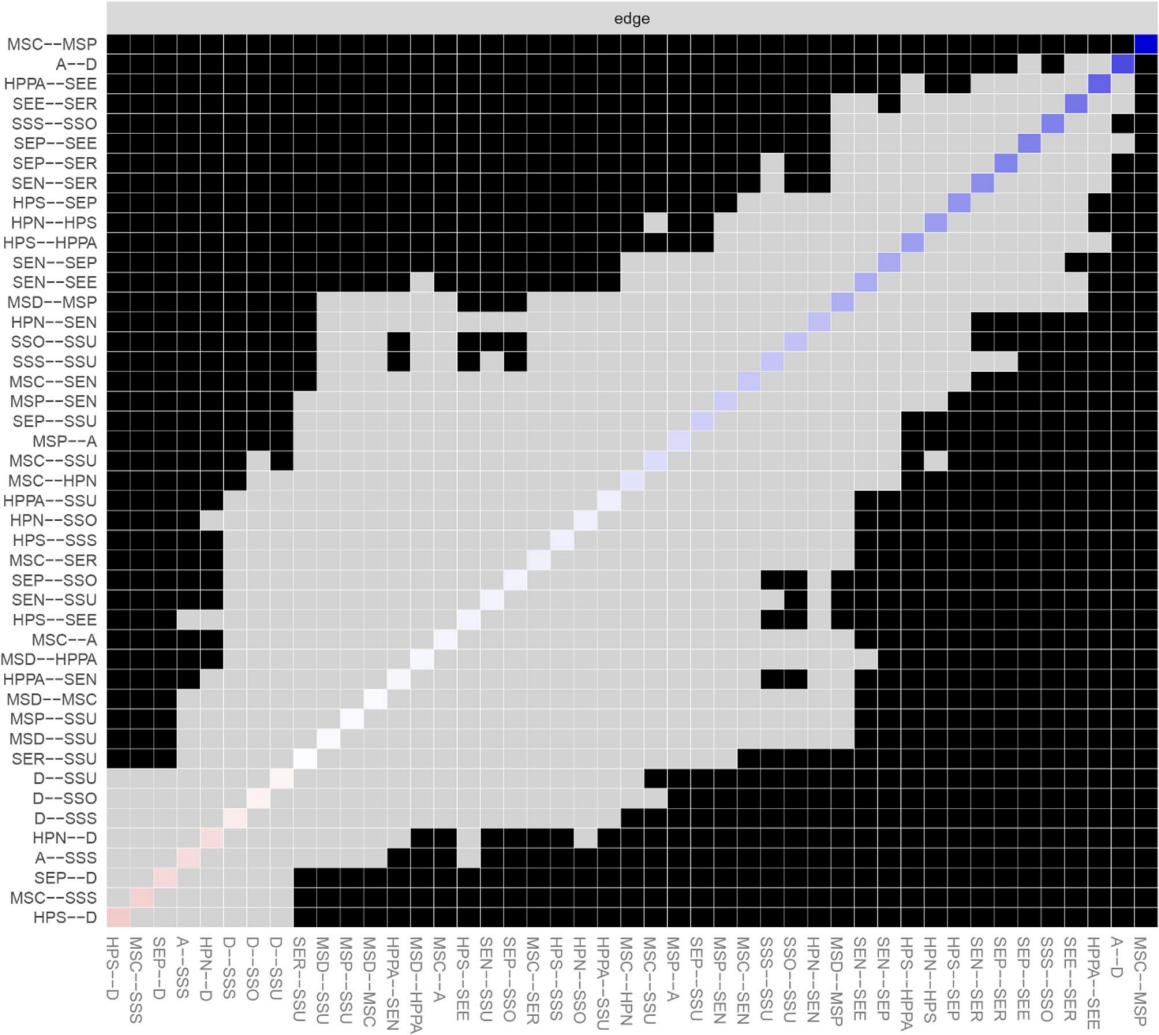
Estimation of edge weight difference by bootstrapped difference test Note: Gray boxes indicate no significant differences between nodes, while black boxes represent nodes that exhibit significant differences (α = 0.05); Blue boxes indicate positive correlations

As shown in Fig. 1, self-efficacy in nutrition (weight = 0.13) had a strong connection with the nutritional domain of health-promoting lifestyles. Self-efficacy for psychological well-being (weight = 0.23) and depression (weight=−0.11) were strongly related to stress management. In addition, self-efficacy in exercise (weight = 0.33) had a strong correlation with physical activity. The centrality analysis revealed that self-efficacy for psychological well-being had the largest values for strength (z-score = 1.58) and closeness (z-score = 1.81), meaning that it is the core node (Fig. 2). It also had the largest value for bridge strength (z-score = 1.16), highlighting its vital role in connecting various factors that affect health-promoting lifestyles (Fig. 3). The bootstrapped CIs were small, which suggested that the network was accurate (Fig. 4). The correlation stability coefficient was 0.751 for strength, 0.438 for closeness, and 0.517 for bridge strength, indicating that the network was stable (Fig. 5). The results of bootstrapped difference tests are presented in Figs. 6 and 7. Figure 6 shows that most node strengths significantly differ from each other. The self-efficacy for psychological well-being node has the greatest strength, which is significantly larger than that of most other nodes. Moreover, as shown in Fig. 7, most edges significantly differ from one another.

## Discussion

This study explored the factors influencing health-promoting lifestyles among young and middle-aged adults with metabolic risk factors. The results showed that age, drinking history, knowledge, and self-efficacy significantly affect dietary behaviors. Social support, depression, and self-efficacy can predict stress management, while self-efficacy also contributes to physical activity. The network analysis demonstrated the crucial role of self-efficacy for psychological well-being in the network of factors affecting health-promoting lifestyles. These findings provide a reference for developing targeted interventions to promote healthy lifestyles in this patient group.

In line with a previous study, this study confirmed the pivotal role of self-efficacy in shaping health-promoting lifestyles among young and middle-aged adults with metabolic risk factors [19]. Notably, whereas this prior work identified the effect of self-efficacy on healthy eating and exercise behaviors, our findings extended this understanding by demonstrating that enhancing self-efficacy is of paramount importance for improving stress management practices in this population. In addition, the network analysis showed that self-efficacy for psychological well-being serves as a core and bridge factor affecting health-promoting lifestyles. Therefore, when designing effective and targeted lifestyle interventions for young and middle-aged adults with metabolic risk factors, it is recommended to prioritize this crucial element, with particular emphasis on bolstering patients’ confidence in promoting psychological well-being.

The present study only revealed that knowledge about metabolic syndrome had significant effects on dietary behaviors among young and middle-aged adults with metabolic risk factors. A better understanding of the condition and the importance of its management contributes to healthier eating patterns such as choosing low-fat and low-sugar foods, increasing intake of vegetables and fruits, and regularly eating breakfast. Healthcare providers can employ suitable strategies to deliver the cardiometabolic information, thereby promoting a balanced diet in this patient group. Considering the non-significant effect observed on exercise and stress management, future investigations are needed to provide additional evidence.

In contrast to prior research suggesting a vital role of social support for dietary behaviors among adults with metabolic syndrome [18], our study found that social support significantly affects participants’ practices in managing stress. Strengthening support networks can help individuals cope with stressful events more effectively [36], and thus, this component should be addressed with appropriate strategies when developing lifestyle interventions for these patients. We also recommend further studies to explore the role of social support for exercise and dietary behaviors in this younger group of patients.

Our findings revealed that depression leads to poor stress management in young and middle-aged adults with metabolic risk factors, aligned with a previous study [22]. Patients with depression often experience feelings of hopelessness, loss of interest, insomnia, and lack of energy, which may prevent them from executing stress management strategies [22, 37]. Addressing depression via medications and psychological interventions may promote stress-coping abilities. More evidence on the effects of depression on other health-promoting lifestyles is warranted.

Additionally, we found that younger patients and those who were regular drinkers exhibited worse dietary behaviors. Special attention should be paid to these subgroups of patients when providing lifestyle modifications, and their needs should be addressed via tailored interventions.

Several limitations should be acknowledged in this study. Firstly, as this study employed a cross-sectional design, the findings warrant further examination through longitudinal and experimental studies. Secondly, the participants were recruited from a highly developed city in China using convenience sampling, which may limit the generalizability of the results. Thirdly, the Cronbach’s alpha values for the Social Support Rating Scale and the Hospital Anxiety and Depression Scale-Depression subscale are relatively low. Lastly, the use of self-reported tools for data collection might have introduced social desirability and recall biases. However, social desirability bias may be mitigated, as participants are more likely to respond truthfully when anonymity and confidentiality are ensured.

## Conclusions

Self-efficacy, knowledge, social support, and depression are indispensable modifiable factors affecting health-promoting lifestyles among young and middle-aged adults with metabolic risk factors. In particular, self-efficacy for psychological well-being represents a key target for developing effective lifestyle interventions.

## Supplementary Information

Supplementary Material 1.

## Data Availability

The data that support the findings of this study are available on request from the corresponding author. The data are not publicly available due to privacy and ethical restrictions.

## Abbreviations

CHC: Community health centers
SD: Standard deviation
VIF: Variance inflation factors
